# Robot-Assisted Laparoscopic Endorectal Pull-Through Combined with Deloyers Turnover in Long-Segment Hirschsprung Disease: A Case Report

**DOI:** 10.1055/a-2640-4118

**Published:** 2025-07-11

**Authors:** Maria Stella Cipriani, Maria G. Faticato, Federica Fanti, Michela C. Y. Wong, Girolamo Mattioli

**Affiliations:** 1 Department of APediatric Surgery, IRCCS, Istituto Giannina Gaslini, Genoa, Italy; 2 Dipartimento di Neuroscienze, Riabilitazione, Oftalmologia, Genetica e Scienze Materno-Infantili, University of Genoa, Genoa, Italy

**Keywords:** Hirschsprung disease, Deloyers technique, robotic surgery, case report

## Abstract

We report the first documented pediatric case of the Deloyers procedure performed using robotic surgery to treat a female patient with long-segment Hirschsprung disease. A 9-month-old child was diagnosed with long-segment Hirschsprung disease. Despite rectal irrigations, the patient experienced refractory constipation and enterocolitis, necessitating exploratory surgery and ileostomy. A transition zone was identified at the proximal transverse colon, with mapping biopsies confirming aganglionosis up to the splenic flexure. At 16 months of age, the child underwent a robot-assisted endorectal pull-through with ileostomy closure. Four 8-mm trocars were placed in the epigastric, left subcostal, right subcostal, and right flank regions. Dissection and mobilization extended distally beyond the peritoneal reflection and proximally to the hepatic flexure, preserving the marginal vascular arcades. The middle and right colic arteries were ligated, while the ileocolic artery was preserved. Indocyanine green fluorescence imaging confirmed adequate vascularization of the hepatic flexure. A counterclockwise 180-degree rotation of the right colon was performed. In the perineal phase, a mucosal incision above the dentate line was followed by progressive mucosectomy to reach the isolated rectum. The colon was pulled through, and a tension-free coloanal anastomosis was performed. The postoperative period was uneventful at the 6-month follow-up. Robot-assisted laparoscopic endorectal pull-through with the Deloyers procedure is a feasible and safe technique for long-segment Hirschsprung disease. Larger case series are required to assess its long-term outcomes and potential advantages. An explanatory video of the surgery is available.

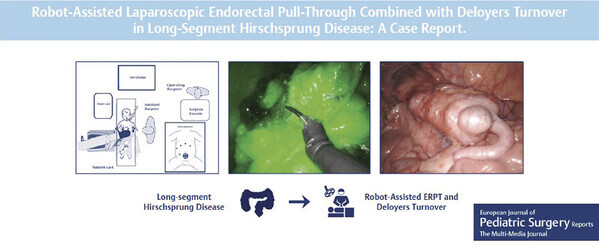

## Introduction

Deloyers' turnover of the right colon to prevent ileal obstruction in long-segment Hirschsprung disease, in combination with endorectal pull-through, has been described as a successful and feasible option in children. 1 This technique differs slightly from the original approach in adult colorectal surgery 2 due to the need for a coloanal anastomosis, which requires preserving the vascular supply up to the proximal transverse colon. 3 In recent years, the use of robotic approaches in pediatric surgery has been growing, demonstrating clear advantages in various procedures. These developments are increasingly being incorporated into the management of complex conditions such as Hirschsprung disease. 4

To our knowledge, this is the first reported case of robot-assisted laparoscopic endorectal pull-through combined with Deloyers turnover in a child with long-segment Hirschsprung disease.

## Case Report

A 9-month-old female infant was diagnosed with long-segment Hirschsprung disease after presenting with severe constipation and signs of intestinal obstruction. She was born in another hospital at 37 weeks' gestation and passed meconium within the first 24 hours. However, after discharge, she developed chronic constipation requiring daily rectal stimulation to facilitate bowel movements. By 9 months of age, she developed toxic megacolon requiring intensive care and was transferred to our center for stabilization and further management. Upon transfer, the patient was clinically stabilized. Rectal suction biopsies confirmed the diagnosis of Hirschsprung disease. She was discharged with instructions to continue rectal irrigations while awaiting definitive reconstructive surgery. A contrast enema was performed, which suggested a long-segment form of the disease.

Despite ongoing rectal irrigations, she experienced refractory constipation and episodes of Hirschsprung-associated enterocolitis, prompting the decision to proceed with exploratory surgery and diverting ileostomy. Intraoperatively, the transition zone was identified at the proximal transverse colon. Mapping biopsies confirmed aganglionosis up to the splenic flexure, consistent with long-segment disease. Furthermore, during the intestinal exploration, an incidental finding of incomplete intestinal rotation and Ladd's bands at the cecal level was observed and subsequently lysed.

**Fig. 1:**
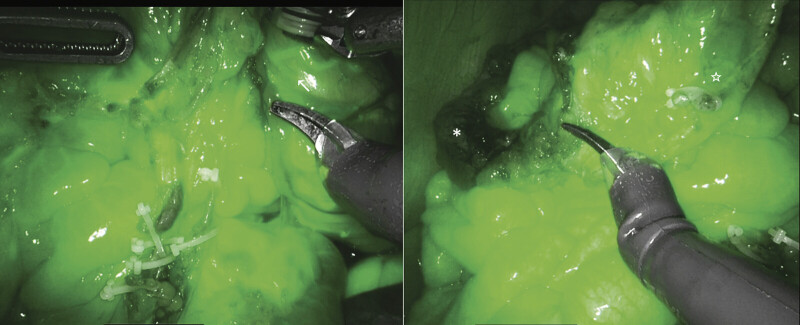
The middle colic artery was clipped and divided proximally, preserving the vascular arcades. The transverse colon was well perfused at the proximal (arrow) and middle (star) levels, but not at the distal (asterisk) level.

**Fig. 2:**
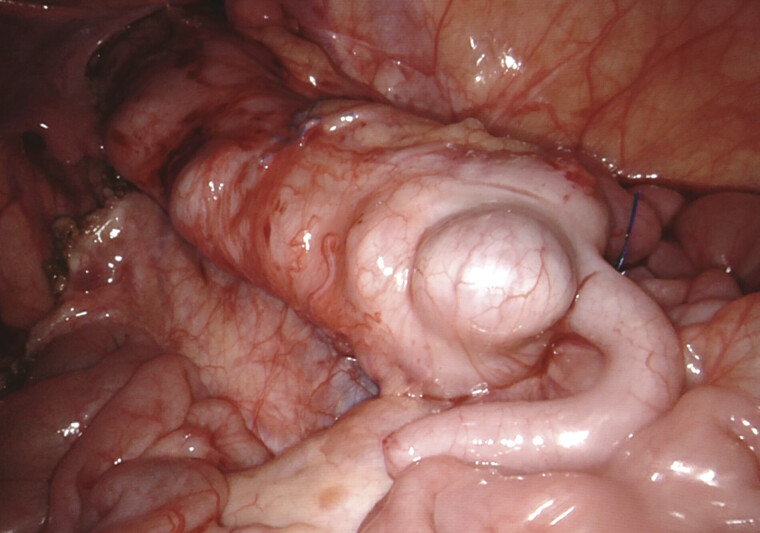
The colon was derotated by 180 degrees counterclockwise using Deloyers' technique.

At 16 months of age, a robot-assisted Swenson pull-through was performed, combined with the Deloyers procedure and ileostomy closure. The robotic surgery system used was the Da Vinci Xi® (Intuitive Surgical Inc., Sunnyvale, CA, USA). The patient's weight at the time of surgery was 9.4 kg. Under general anesthesia, four 8-mm robotic trocars were placed in the epigastric region, left and right subcostal areas, and right flank. Robotic dissection and mobilization were performed distally beyond the peritoneal reflection and proximally up to the hepatic flexure. Distally, dissection was performed in the Swenson plane, circumferentially around the rectum, with meticulous attention to remain close to the bowel wall. Proximal dissection, from the middle transverse colon to the entire right colon, was performed with preservation of the marginal vascular arcade. The middle and right colic arteries were selectively ligated, while the ileocolic artery was preserved. Vascular perfusion of the colonic segment was assessed intraoperatively using indocyanine green fluorescence (ICG) imaging, confirming adequate vascularization of the hepatic flexure ( Fig. 1 ). Following this, the right colon was mobilized and derotated 180 degrees counterclockwise according to the Deloyers procedure ( Fig. 2 ). The robotic console time for the abdominal phase lasted 57 minutes. During the perineal phase, a circumferential mucosal incision was made just above the dentate line. Progressive mucosectomy was performed to reach the isolated rectum. The previously mobilized colonic segment was pulled through to the anal canal, and a tension-free coloanal anastomosis was performed. The ileostomy was closed during the same procedure. The postoperative course was uneventful. The patient tolerated reintroduction of oral feeding and demonstrated regular bowel function. She had a hospital stay of 7 days. At 1 month postoperatively, a sedation-assisted examination with calibration of the coloanal anastomosis revealed a patent, wide, and elastic anastomosis. At the 6-month follow-up, the child had regular bowel movements without the need for rectal irrigations, was thriving with adequate weight gain, and showed no clinical signs of postoperative complications.

## Discussion and Conclusion

**Video 1** The video illustrates the key steps of the procedure: distal dissection in the Swenson plane, proximal dissection with preservation of the marginal vascular arcade, ICG fluorescence assessment of hepatic flexure perfusion, Deloyers' Turnover, endorectal pull-through, and final check of a tension-free coloanal anastomosis.


This case report demonstrates the feasibility of a robotic Deloyers maneuver and endorectal pull-through in a 16-month-old child with long-segment Hirschsprung disease, along with the use of ICG imaging to assess the adequate vascularization of the anastomosis.

Current guidelines for Hirschsprung disease do not clearly recommend the optimal pull-through technique, nor do they mention turnover techniques. 5 6 Deloyers, in 1958, described a method to mobilize the proximal transverse colon and perform a colorectal anastomosis proximal to the peritoneal reflection in adult patients. 2 This is achieved by performing a counterclockwise 180-degree rotation of the right colon after ligating the middle and right colic arteries while preserving the ileocolic artery. This technique has also been applied to long-segment Hirschsprung disease. However, in these cases, the colon needs to be mobilized more distally than initially described by Deloyers, as it must cross the pelvis to reach the anal canal for a successful coloanal anastomosis, which can present significant challenges. Elhalaby et al addressed these issues, highlighting the difficulties of applying the technique to long-segment Hirschsprung disease. 3 A case series has also shown the use of the Deloyers and Turnbull maneuvers not only for long-segment disease but also in cases where the marginal artery or Drummond's artery had been sacrificed during transverse colostomy creation or redo-surgery due to extensive stenosis of the pulled-through colon. In this series, the Deloyers procedure was used when performing a proximal transverse colon pull-through, with positive outcomes. 1 A systematic review revealed that robotic colorectal surgery is a safe and effective approach in children, particularly for complex conditions such as Hirschsprung disease, inflammatory bowel diseases, and anorectal malformations. 7 Zhang et al, in a retrospective study, compared robotic and laparoscopic-assisted Soave pull-through surgeries, demonstrating that both techniques are feasible and effective at all ages, with comparable postoperative complication rates and medium-term bowel functional outcomes. 8 The robotic-assisted Deloyers procedure has been described in an adult colorectal cancer, utilizing ICG imaging to confirm proper perfusion of the ascending colon after preserving the marginal vascular arteries and the ileocolic artery. 9

This case report represents the first robotic-assisted Deloyers procedure and endorectal pull-through in a child. The right colon turnover was technically easier to perform with robotic assistance, thanks to the 360-degree range of movement and 3D visualization, which enhanced mesocolon visibility and allowed for easier manipulation of the isolated colon. Additionally, the enhanced dissection of the rectum made possible by robotic surgery provided optimal conditions for performing a transanal coloanal anastomosis.

As pediatric robotic surgery continues to evolve, we believe this case may serve as a promising indication for the use of robotic technology in similar procedures. An explanatory Video 1 of the surgery is available.

## Notes

Importance for Pediatric Surgeon This article describes the first documented case of laparoscopic endorectal pull-through associated with Deloyers procedure performed using robotic surgery to treat a child with long-segment Hirschsprung disease.
